# Exome sequencing circumvents missing clinical data and identifies a *BSCL2* mutation in congenital lipodystrophy

**DOI:** 10.1186/1471-2350-15-71

**Published:** 2014-06-24

**Authors:** Jens Schuster, Tahir Naeem Khan, Muhammad Tariq, Pakeeza Arzoo Shaiq, Katrin Mäbert, Shahid Mahmood Baig, Joakim Klar

**Affiliations:** 1 Department of Immunology, Genetics and Pathology, Science for Life Laboratory, Uppsala, Sweden; 2 Human Molecular Genetics Laboratory, National Institute for Biotechnology and Genetic Engineering (NIBGE), Faisalabad, Pakistan

**Keywords:** Exome sequencing, Lipodystrophy, BSCL2

## Abstract

**Background:**

Exome sequencing has become more and more affordable and the technique has emerged as an important diagnostic tool for monogenic disorders at early stages of investigations, in particular when clinical information is limited or unspecific as well as in cases of genetic heterogeneity.

**Methods:**

We identified a consanguineous Pakistani family segregating an autosomal recessive phenotype characterized by muscular hypertrophy, mild mental retardation and skeletal abnormalities. The available clinical information was incomplete and we applied whole exome sequencing in an affected family member for the identification of candidate gene variants.

**Results:**

Exome sequencing identified a previously unreported homozygous mutation in the acceptor splice site of intron 5 in the *BSCL2* gene (c.574-2A > G). Expression analysis revealed that the mutation was associated with skipping of exon 6. *BSCL2* mutations are associated with Berardinelli-Seip congenital lipodystrophy and a clinical re-evaluation of affected individuals confirmed the diagnosis.

**Conclusions:**

Exome sequencing is a powerful technique for the identification of candidate gene variants in Mendelian traits. We applied this technique on a single individual affected by a likely autosomal recessive disorder without access to complete clinical details. A homozygous and truncating mutation was identified in the *BSCL2* gene suggesting congenital generalized lipodystrophy. Incomplete phenotypic delineations are frequent limiting factors in search for a diagnosis and may lead to inappropriate care and follow-up. Our study exemplifies exome sequencing as a powerful diagnostic tool in Mendelian disorders that may complement missing clinical information and accelerate clinical diagnosis.

## Background

Mutation detection in genetic diseases is important for a definite diagnosis for genetic counseling, future prenatal diagnosis and therapy. Next generation sequencing technologies are increasingly being used as a diagnostic tool for genetic disorders and have been shown particularly successful in genetically heterogeneous phenotypes, or when ascertainment of clinical features and symptoms are unattainable [1-10]. Furthermore, exome sequencing is in many cases a cost-effective diagnostic method of patients with Mendelian traits compared to the interrogation of candidate genes one by one.

We report herein on a family segregating short and muscular stature, mild mental retardation and acromegalic appearance as an autosomal recessive trait. The clinical information was otherwise sparse. Using whole exome sequencing (WES), we identified a homozygous mutation in the acceptor splice site of intron 5 of the *BSCL2* gene previously associated to congenital generalized lipodystrophy type 2 (CGL2) [11]. Functional analysis revealed that the mutation results in a complete skipping of exon 6 leading to a predicted change in reading frame and an early termination of the protein. Clinical re-evaluation of the patients confirmed the diagnosis congenital generalized lipodystrophy.

## Methods

### Study subjects

**Figure 1:**
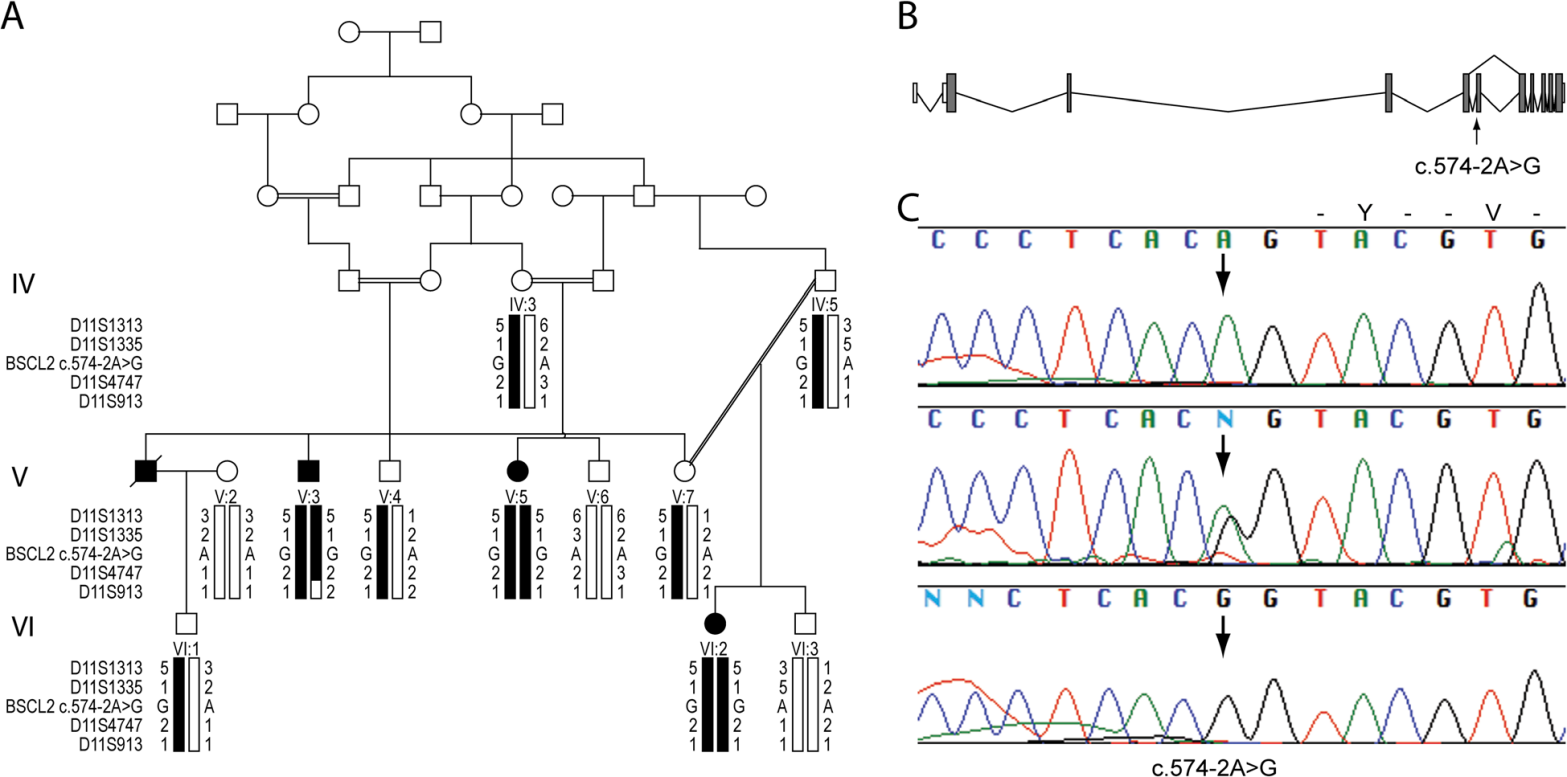
**Pedigree of family segregating Berardinelli-Seip congenital lipodystrophy and a *BSCL2 *splice site mutation. A)** Family structure indicating consanguinity and autosomal recessive inheritance. Haplotypes spanning the *BSCL2* gene mutation (c.574-2A > G) on chromosome 11 are shown below each symbol. The mutation segregates within a shared homozygous (autozygous) region in all affected individuals. **B)** The variant is predicted to lead to an exon skipping of exon 6, which would results in a frameshift and premature termination of the protein (p.Y256fsX48). **C)** Chromatogram showing cDNA sequences obtained from expressed mini-gene constructs containing *BSCL1* gene spanning exons 5–7, with or without the variant. The variant is situated within the acceptor splice site of intron 5 of the *BSCL2* gene. Wild-type (w.t.) *BSCL1* construct (top), co-transfection of w.t. and mutant construct (middle) and mutated construct (bottom).

We identified a consanguineous six generation Pakistani pedigree with three members, two females and one male, who presented with similar features. The pedigree structure suggested an autosomal recessive inheritance (Figure 1A). The family lived in an isolated rural area and the initial clinical data was scanty with information on muscular hypertrophy, mild mental retardation, and possible skeletal malformations. The diagnosis remained unclear and further clinical information and laboratory data were unavailable. Informed consent was obtained from all individuals who participated in this study or their legal guardian under a protocol approved by the local ethical committee at National Institute for Biotechnology and Genetic Engineering (NIBGE), Faisalabad, Pakistan. This includes consent from the patients to publish images.

### Exome sequencing and mutation detection

DNA from an affected individual (V3) was sonicated using a Covaris S2 instrument (Covaris, Inc., Woburn, MA, USA). Fragment libraries were created from the sheared samples using the AB Library Builder System (Life Technologies, Carlsbad, California, USA) and target enrichment was performed using the Agilent SureSelect Human All Exon v4 kit according to the manufacturer's protocols (Agilent, Santa Clara, CA, USA). Exome capture was conducted by hybridizing the DNA libraries with biotinylated RNA baits for 24 h followed by extraction using streptavidin coated magnetic beads. Captured DNA was then amplified followed by emulsion PCR using the EZ Bead System (Life Technologies) and sequenced on the SOLiD5500xl system, generating over 100 million reads of 75 bp length for each of the samples. Alignment of reads to the human reference sequence (hg19 assembly) and variant detection was performed using v2.1 of the LifeScope Software (Life Technologies). SNPs and indel data was stored in an in-house exome database together with variant annotation information obtained from ANNOVAR [12] and dbSNP137. Custom R scripts were used to identify potentially damaging variants not present in any of ~800 exomes from our in-house database. A minimal sequence depth of x5 was used as a quality cut-off. The *BSCL2* variant was confirmed by direct sequencing with dideoxy chain-termination method (Applied Biosystems BigDye Terminator v3.1 Cycle Sequencing Kit) on a 3730xl DNA Analyzer (Applied Biosystems, Foster City, CA). Sequence analysis was performed using the Sequencer software (Gene Codes Corporation, Ann Arbor, MI, USA).

### Minigene expression construct

In total, 5 μg of genomic DNA from a heterozygous individual (VI:1) was used for amplification with primers *BSCL2* e5F (GTG ATG CTG CAT TAC CGC TCA GAC) and *BSCL2* e7R (CTG CAA AGA GAA GCG GTG TCG G). The PCR product was re-amplified using primers *BSCL2* e5F Hind III (AGC TCA AGC TTC GTG ATG CTG CAT TAC CGC TCA GAC) and *BSCL2* e7R Kpn I (CCC GCG GTA CCG CTG CAA AGA GAA GCG GTG TCG G) to introduce restriction sites. The resulting PCR product was cloned into a TA cloning vector (Invitrogen, Carlsbad, CA, USA) and individual clones were analyzed by Sanger sequencing. Clones representing the two alleles (wt allele (ag) and mutant allele (gg)) were identified and subsequently inserted into a pEGFP-C2 expression vector (Clontech, Mountain View, CA, USA) using Hind III, Kpn I and Rapid DNA ligation kit (Fermentas, Thermo Fisher Scientific, Waltham, MA, USA) and confirmed by sequencing. Thus, the minigene was fused to green fluorescent protein (Additional file 1: Figure S1A).

### Splicing assay

The two minigene expression constructs were transfected into HEK293T cells using FuGene® Transfection reagent following manufacturer’s recommendations (Promega, Fitchburg, WI, USA). Cells were grown in DMEM supplemented with 10% fetal calf serum, 2 mM L-glutamine, 20 IU penicillin/streptomycin and non-essential amino acids (all Sigma-Aldrich, St. Louis, MI, USA) at 37°C with 5% CO_2_ in a humidified atmosphere. Expression of the GFP-minigene was confirmed by fluorescence microscopy after 48 hours. Subsequently, cells were harvested and total RNA was isolated using TRIZOL^TM^ (Invitrogen), following the manufacturer’s protocols. Splicing of the minigene was detected by reverse transcription of 2 μg of total RNA using oligo-d(T) primers, utilizing the VILO cDNA synthesis kit (Invitrogen). Splicing products were amplified by standard PCR with primers Assay Forward (TAC AAG TCC GGC CGG ACT CAG ATC) and *BSCL2* e7R (Additional file 1: Figure S1B). PCR products were cloned into a TA cloning vector (Invitrogen), and individual clones were analyzed by Sanger sequencing.

## Results

### Genetic analysis

Targeted enrichment of DNA from one affected family member, followed by WES and filtering, identified 34 homozygous variants, including one in the *BSCL2* gene (NG_008461.1; NM_032667.6) (Additional file 2: Table S1). The sequencing resulted in 97% target base coverage (>1X) and 85% of the target bases were covered >20X. The variant is situated in the acceptor splice site of intron 5 (c.574-2A > G) of the gene and predicts skipping of exon 6 with a frameshift and premature termination codon (p.Y256fsX48) (Figure 1B). Sanger sequencing confirmed homozygosity for the c.574-2A > G variant in all three affected members (V:3, V:5 and VI:2) whereas three available parents were heterozygous (Figure 1A,C). Furthermore, the variant was excluded on 200 Swedish and 200 Pakistani control chromosomes and is not present in 6503 exomes from the Exome Variant Server, NHLBI GO Exome Sequencing Project (ESP), Seattle, WA (URL: http://evs.gs.washington.edu/EVS/). Segregation of a homozygous region surrounding the *BSCL2* variant was confirmed using microsatellite markers (Figure 1A).

### Study subjects

**Figure 2:**
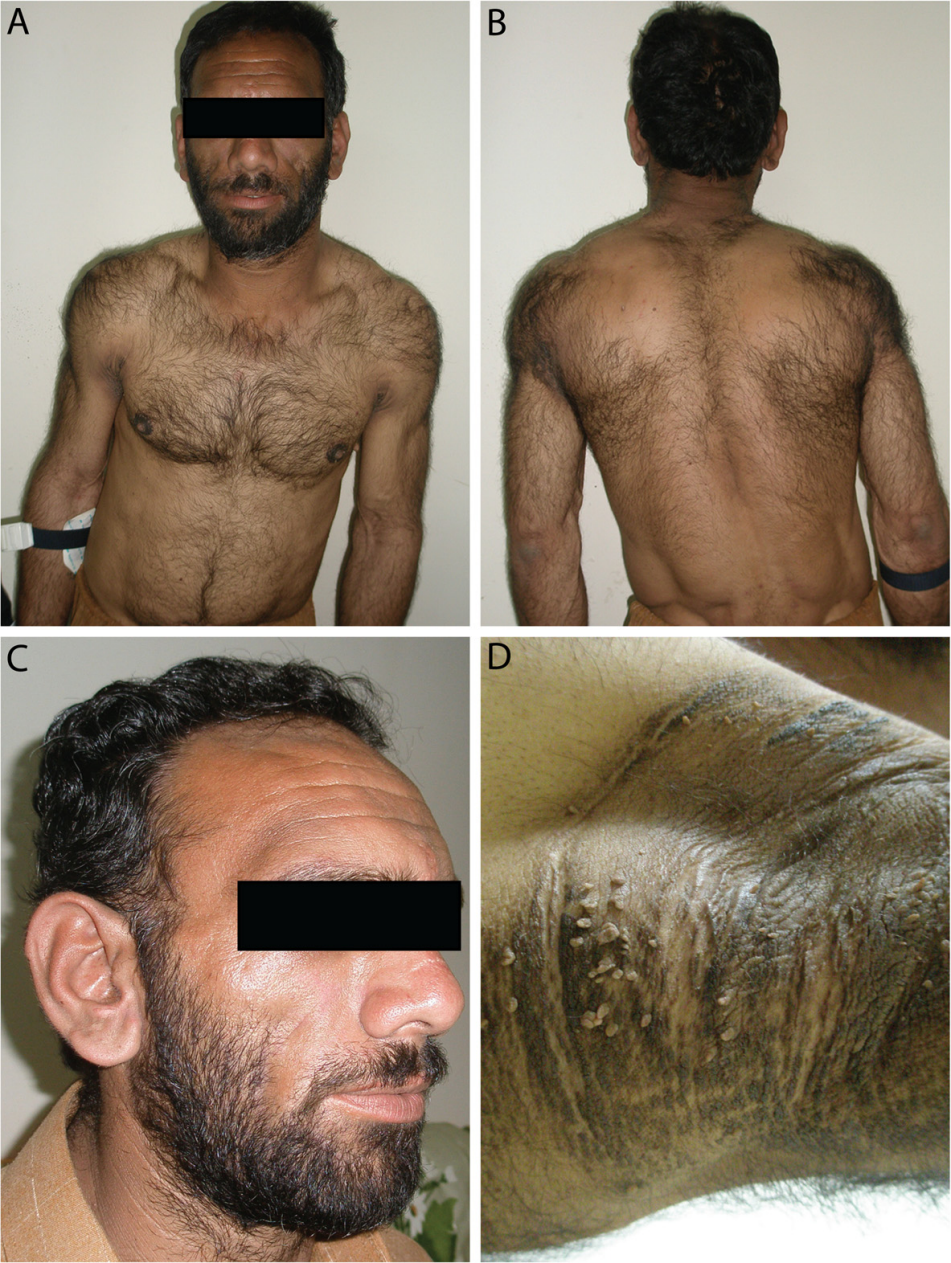
**Clinical features of the patients. A-C)** The affected family member V:3 illustrating muscular stature **(A, B)** acromegalic appearance **(C)** and axillary acanthosis nigricans **(D).**

The family was revisited after WES analysis for a clinical re-evaluation of the three affected family members. The investigation revealed generalized lipodystrophy, axillary acanthosis nigricans relatively large hands and feet, muscular hypertrophy and acromegaloid appearance (Figure 2). The three affected individuals had mental retardation and individual V:5 had type 2 diabetes and a spastic gait suggesting upper motor neuron involvement. None of the three affected members had signs of muscle weakness. Hearing was normal. The proband (V:3) was available for ultrasound investigation and biochemical analysis. Ultrasound of abdomen showed enlarged liver (16.5 cm) and spleen (13.5 cm). Biochemical analysis of serum revealed increased levels of alanine aminotransferase levels (ALT; 64 U/L) and increased alkaline phosphatase levels (328 U/L). Unexpectedly, S-triglyceride and cholesterol levels were within the upper normal range as well as fasting glucose levels. The combined findings from our re-investigation of the family confirmed congenital generalized lipodystrophy.

### Functional analysis

To evaluate the predicted effects of the acceptor splice site mutation in intron 5 of the *BSCL2* gene, we created a mini-gene consisting of exon 5 through exon 7 including the two introns, fused to green fluorescent protein (Additional file 1: Figure S1A). Mini-genes containing the wild-type (wt; ag) and mutated (gg) splice site variants, respectively, were expressed in HEK293T cells and RNA was isolated 48 h after transfection. The amplified spliced products were analyzed by agarose gel electrophoresis and we observed a band of expected size from the wt construct (420 bp) whereas a shorter product (322 bp) was generated when amplifying from the mutated construct (Additional file 1: Figure S1B). The PCR products were cloned and analyzed by sequencing that confirmed the predicted skipping of exon 6 (Additional file 1: Figure S1C).

## Discussion

We identified a consanguineous family segregating autosomal recessive muscular hypertrophy mild mental retardation and acromegaloid appearance in three individuals. Detailed clinical and biochemical data were unavailable and we decided to perform WES on a single affected family members in search for homozygous and potentially damaging gene variants. Analysis of WES data showed a homozygous acceptor splice site mutation, c.574-2A > G, in intron 5 of the *BSCL2* gene. The *BSCL2* gene is associated with congenital generalized lipodystrophy type 2 (CGL2) and, indeed, clinical re-evaluation of our patients confirmed characteristic clinical features of the disease in all three affected members [11]. Taken together the deleteriousness of the variant in *BSCL2* and the clinical overlap with the preliminary information we had of the family, this was considered the most likely candidate variant of the ones indentified. Furthermore, we could by using a mini-gene construct spanning exons 5–7 of the mutated *BSCL2* allele show that the mutation results in skipping of exon 6, an altered reading frame and a predicted early termination of the protein. Individuals with CGL2 can usually be recognized at birth, or soon thereafter, due to a near-total lack of body fat and prominent muscularity. Later characteristics include insulin resistance, hypertriglyceridemia, hepatic steatosis and early onset of diabetes. A high prevalence of mild to moderate intellectual impairment has been observed and patients have a higher risk for premature mortality, mainly due to cardiac failure. Additional features include acanthosis nigricans, hepatomegaly, hyperandrogenism and skeletal muscular hypertrophy [13,14]. The disorder may, however, be mistaken for other types of lipodystrophies, especially in adult stage, making a clear diagnosis problematic [11,15-18]. Considerable efforts have been made in the characterization and identification of the molecular basis of inherited lipodystrophies, and to date at least 11 gene-loci are known [13].

In our family, the initial information suggested a disabling condition segregating as an autosomal recessive trait. The clinical data were insufficient for a diagnosis and further investigations of the family were impossible within a reasonable time-frame. Thus, we decided to apply WES and, from our findings, we were able to suggest that the patients were affected by CGL2 within a few weeks from the sampling of DNA. Much later, the diagnosis was confirmed through directed clinical and biochemical investigations of the family. Moreover, this exemplifies a time-saving aspect of exome sequencing in situations of diagnostic challenges, inaccessible clinical data as well as acute illnesses [13]. Furthermore, rare diseases such as CGL2 may remain undiagnosed due to socio-economic constraints and inaccessible health care. Under such circumstances exome sequencing may become an attractive diagnostic complement with a continuous reduction in price. In a clinical setting, an alternative to WES could be the use of targeted sequencing of a specific subset of genes responsible for a certain condition. Although we are limited to clinically available disease-targeted enrichment tests, the resulting data generally have a much higher or often complete coverage of the targets [19].

Taken together, our results show the usefulness of WES for the identification of a single homozygou*s BSCL2* mutation in a family segregating an autosomal recessive trait. The mutation was analyzed in a cell-based assay that confirmed exon skipping. Using the findings from WES we could suggest the diagnosis of CGL2 in advance of complete clinical information from the patients. The use of exome sequencing accelerated and facilitated the subsequent and confirming investigations, with implications for care and follow-up of affected individuals.

## Conclusions

This study illustrates how whole exome sequencing (WES) can be used in a clinical setting to identify gene mutations in search for a specific diagnosis. We show that WES can be used to accelerate clinical investigations of heterogeneous Mendelian traits for yet inconclusive cases.

## Abbreviations

BSCL: Berardinelli-Seip congenital lipodystrophy; CGL: congenital generalized lipodystrophy.

## Competing interests

The authors declare that they have no competing interests.

## Authors’ contributions

JS helped in the design of the study, carried out the functional analyses and helped to draft the manuscript. TNK, MT, PA and KM carried out the molecular genetic studies. SMB and TNK carried out the clinical evaluation of the patients. JK designed the study and drafted the manuscript. All authors read and approved the final manuscript.

## Pre-publication history

The pre-publication history for this paper can be accessed here:

http://www.biomedcentral.com/1471-2350/15/71/prepub

## Supplementary Material

Additional file 1: Figure S1 **A)** Minigene construct consisting of exon 5 through to exon 7 of the *BSCL2* gene, including the introns, fused to green fluorescent protein (GFP). Two minigenes representing wt (ag) and mutated (gg) splice site were used. **B)** PCR based assay of the minigenes. An observed band of expected size in the wt construct (420 bp), but a smaller product (322 bp) corresponding to the expected exon skipping could be identified from the mutated construct. **C)** Sanger sequencing of cDNA from mutated (gg) construct confirms skipping of exon 6. Click here for file

Additional file 2: Table S1 Homozygous variants identified by WES. *snp137 indicates if the variant is present in the 137 version of dbSNP. Note that presented variants, besides the identified variant in the BSCL2 gene, are not confirmed by Sanger sequencing, and could thereby possibly be sequencing errors. Click here for file
