# Supplementary figures and images for: Exome sequencing circumvents missing clinical data and identifies a BSCL2 mutation in congenital lipodystrophy
Source: BMC Med Genet. 2014 Jun 24;15:71. doi: 10.1186/1471-2350-15-71 (PMC4076434; doi:10.1186/1471-2350-15-71)

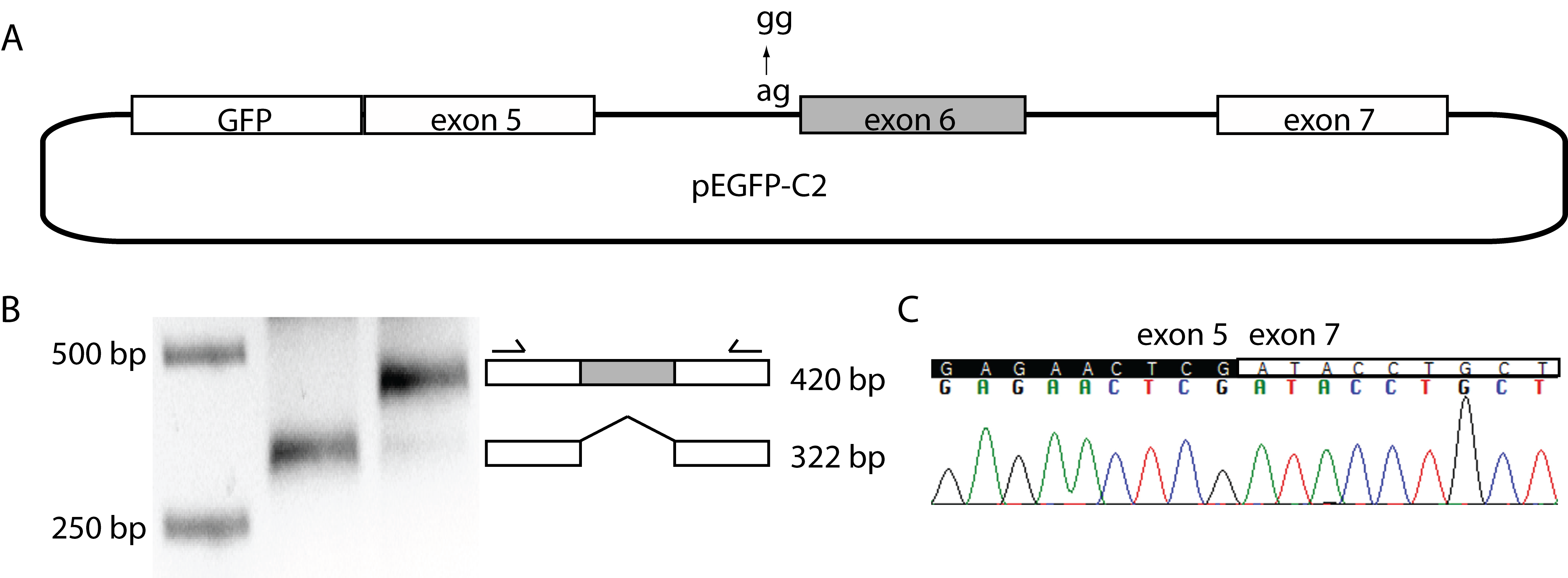

Supplement: Additional file 1: Figure S1 — A) Minigene construct consisting of exon 5 through to exon 7 of the BSCL2 gene, including the introns, fused to green fluorescent protein (GFP). Two minigenes representing wt (ag) and mutated (gg) splice site were used. B) PCR based assay of the minigenes. An observed band of expected size in the wt construct (420 bp), but a smaller product (322 bp) corresponding to the expected exon skipping could be identified from the mutated construct. C) Sanger sequencing of cDNA from mutated (gg) construct confirms skipping of exon 6. [file 1471-2350-15-71-S1.png]
