# Supplementary material for: Exome sequencing circumvents missing clinical data and identifies a BSCL2 mutation in congenital lipodystrophy
Source: BMC Med Genet. 2014 Jun 24;15:71. doi: 10.1186/1471-2350-15-71 (PMC4076434; doi:10.1186/1471-2350-15-71)
Supplement: Additional file 2: Table S1 — Homozygous variants identified by WES. *snp137 indicates if the variant is present in the 137 version of dbSNP. Note that presented variants, besides the identified variant in the BSCL2 gene, are not confirmed by Sanger sequencing, and could thereby possibly be sequencing errors. [file 1471-2350-15-71-S2.docx]

| *********** CanvasDB filtering analysis report ****************** | | | |
| --- | --- | --- | --- |
| ** Analysis of recessive variants in samples: ugc_414_4 | | | |
| ** Variants allowed to be detected in at most 8 (1%) of other samples | | | |
| ********************************************************** | | | |
| ------------------- Homozygous SNPs --------------------- | | |  |
| **SNP_id** | **class** | **snp137*** | **gene** |
| chr1\|145328407\|G\|C | nonsynonymous |  | NBPF10 |
| chr1\|90484266\|A\|G | nonsynonymous |  | ZNF326 |
| chr3\|120371474\|G\|T | nonsynonymous |  | HGD |
| chr3\|167762617\|T\|C | nonsynonymous |  | GOLIM4 |
| chr3\|183860839\|G\|T | splicing |  | EIF2B5 |
| chr3\|195452136\|C\|T | nonsynonymous | rs75979959 | MUC20 |
| chr7\|116397489\|A\|G | splicing |  | MET |
| chr10\|111886249\|T\|A | nonsynonymous | rs143966199 | ADD3 |
| chr10\|26357747\|C\|A | stopgain |  | MYO3A |
| chr10\|5014457\|T\|G | nonsynonymous |  | AKR1C1 |
| chr11\|27679997\|T\|C | nonsynonymous |  | BDNF |
| chr11\|56344851\|G\|A | nonsynonymous |  | OR5M10 |
| chr11\|60233592\|T\|C | nonsynonymous | rs193204741 | MS4A1 |
| chr11\|62291778\|C\|A | nonsynonymous |  | AHNAK |
| chr11\|62459947\|T\|C | splicing |  | BSCL2 |
| chr11\|64517959\|G\|A | nonsynonymous |  | PYGM |
| chr11\|72537606\|T\|C | nonsynonymous |  | ATG16L2 |
| chr15\|30665283\|G\|C | nonsynonymous |  | CHRFAM7A |
| chr15\|85400377\|A\|G | nonsynonymous |  | ALPK3 |
| chr16\|20476960\|C\|T | nonsynonymous |  | ACSM2A |
| chr16\|31422740\|G\|T | nonsynonymous |  | ITGAD |
| chr17\|11711084\|G\|T | nonsynonymous |  | DNAH9 |
| chr17\|7727578\|A\|C | nonsynonymous |  | DNAH2 |
| chr17\|79687174\|A\|G | nonsynonymous |  | SLC25A10 |
| chr18\|34324092\|T\|A | nonsynonymous |  | FHOD3 |
| chr19\|10489039\|A\|G | nonsynonymous | rs144960992 | TYK2 |
| chr19\|35551035\|A\|G | nonsynonymous |  | HPN |
| chr19\|55341565\|A\|T | nonsynonymous |  | KIR3DL1 |
| chr2\|69746363\|T\|A | nonsynonymous |  | AAK1 |
| chr20\|13752052\|G\|A | nonsynonymous |  | ESF1 |
| chr20\|20585894\|C\|T | nonsynonymous | rs142419151 | RALGAPA2 |
| chrX\|109694633\|C\|T | nonsynonymous | rs148567845 | RGAG1 |
| chrX\|14932707\|A\|G | nonsynonymous |  | MOSPD2 |
| chrX\|35821038\|T\|G | nonsynonymous |  | MAGEB16 |

**Additional file 2: Table S1.** Homozygous variants identified by WES. *snp137 indicates if the variant is present in the 137 version of dbSNP. Note that presented variants, besides the identified variant in the *BSCL2* gene, are not confirmed by Sanger sequencing, and could thereby possibly be sequencing errors.
